# Supplementary material for: Microevolution of Neisseria lactamica during nasopharyngeal colonisation induced by controlled human infection
Source: Nat Commun. 2018 Nov 12;9:4753. doi: 10.1038/s41467-018-07235-5 (PMC6232127; doi:10.1038/s41467-018-07235-5)
Supplement: Supplementary file 1 — Supplementary Information [file 41467_2018_7235_MOESM1_ESM.pdf]

Supplemental Figure 1: Mutation summary of the *in vivo* versus *in vitro* sample sets.

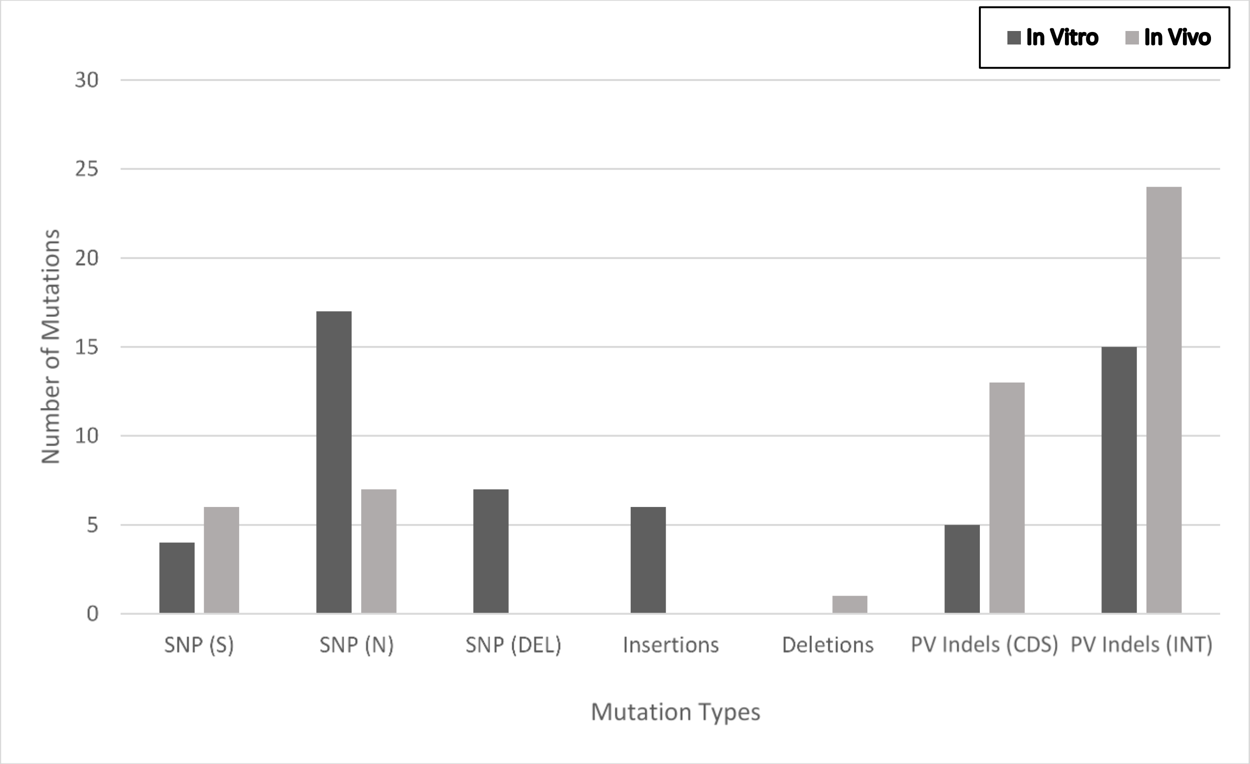

Single nucleotide polymorphisms (SNPs) were classified according to their effect: synonymous (S), non-synonymous (N) and introducing premature stop codons (DEL). Insertion and deletion events (Indels) were examined separately to those occurring in phase variable (PV) homopolymeric tracts. The location of Phase variable "PV" indels were discriminated by those occurring within the coding sequence (CDS) and intergenic regions (INT).

Locus	Annotation	N.M Week 0	N.M Week 2	N.L Week 2	N.L Week 4	N.L Week 16	N.M Week 26
**NEIS0071**	putative lipoprotein (1019bp)	18	18	18	18	284	18
	SNP difference between alleles	32					
		N.M Week 0	N.M Week 2	N.L Week 2	N.L Week 4	N.L Week 16	N.M Week 26
**NEIS0072**	hypothetical protein/NMB0087 (220bp)	8	8	8	8	27	8
	SNP difference between alleles	2					
		N.M Week 0	N.M Week 2	N.L Week 2	N.L Week 4	N.L Week 16	N.M Week 26
**NEIS0899 (lst)**	LOS alpha-2,3-sialyltransferase (1043bp)	82	82	82	82	127	82
	SNP difference between alleles	29					
		N.M Week 0	N.M Week 2	N.L Week 2	N.L Week 4	N.L Week 16	N.M Week 26
**NEIS2514**	hypothetical protein (295bp)	28	28	28	28	48	28
	SNP difference between alleles	28					
		N.M Week 0	N.M Week 2	N.L Week 2	N.L Week 4	N.L Week 16	N.M Week 26
**NEIS1834**	acyl carrier protein reductase (797bp)	84	84	305	305	84	84
	SNP difference between alleles	19					
		N.M Week 0	N.M Week 2	N.L Week 2	N.L Week 4	N.L Week 16	N.M Week 26
**NEIS1835**	N-succinyltransferase (825bp)	26	26	347	347	26	26
	SNP difference between alleles	35					

Supplemental Figure 2: Loci undergoing recombination in volunteer 291 with SNP differences between exchanged alleles. Allele cells are coloured (early timepoint allele) blue and purple (later time point allele) to identify them as distinct for each new allele discovered for the locus. The values in the “SNP difference between alleles” denote the number of variant positions found when contrasting the early timepoint allele to the late timepoint allele. N.M refers to Neisseria meningitidis; N.L are Neisseria lactamica.

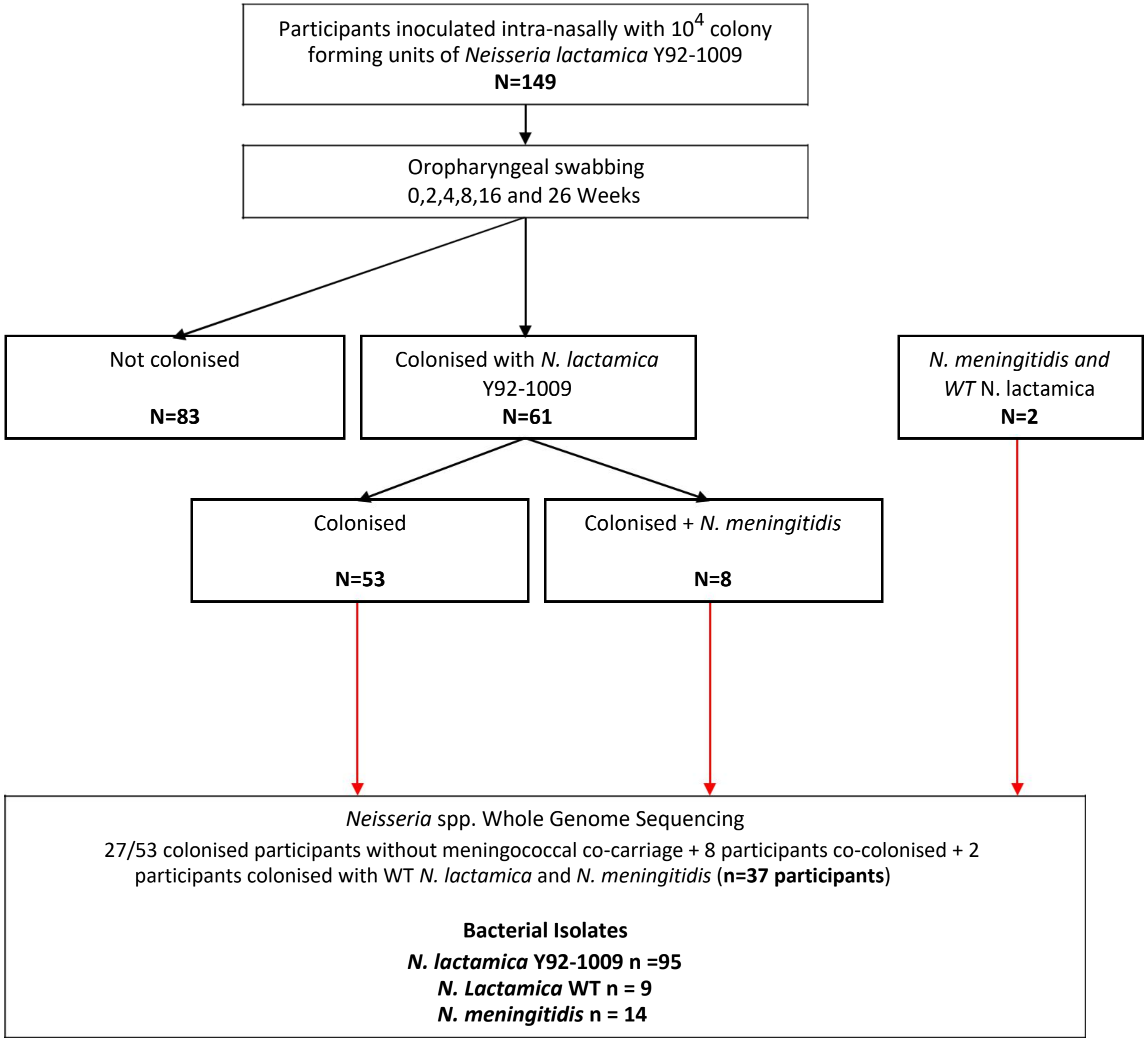

Supplemental Figure 3: Consort Diagram displaying number of isolates (n) sequenced from number of volunteers (N)

Supplementary Table 1: Co-colonised volunteers in long term study

Volunteer	Week 0	Week 2	Week 4	Week 8	Week 16	Week 26	*N. lactamica*	ST
**64**					**839**	**839**	I	613
**77**					**1423**	**1423**	I	613
**104**	**1655**						I	613
**181**						**2394**	I	613
**221**	**466**				**466**		I	613
**255**	**10478**				**213**	**213**	I	613
**279**	**60**					**60**	I	613
**36**	**10457**	**10457**				**10457**	WT	4192
**291**	**41**	**41**				**41**	WT	11524

This table is ordered numerically starting with the volunteers inoculated (I) with *N. lactamica* Y92-1009, (64, 77, 104, 181, 221, 255 and 279) and co-colonised with meningococci. The table concludes by displaying volunteers 36 and 291 co-colonised with wild type (WT) *N. lactamica* and *N. meningitidis*. Key: Dark green blocks- sequenced *N. lactamica* isolates; light green blocks - *N. lactamica* which were not sequenced; red blocks *N. meningitidis* only i.e. no *N. lactamica* co-colonisation; purple - both *N. meningitidis* and *N. lactamica* isolated; white **-** no recovered isolates. The numbers within the red and purple coloured blocks indicate the sequence type (ST) of meningococcal isolates. The *N. lactamica* ST's are listed in the final column.
