# Supplementary material for: Microevolution of Neisseria lactamica during nasopharyngeal colonisation induced by controlled human infection
Source: Nat Commun. 2018 Nov 12;9:4753. doi: 10.1038/s41467-018-07235-5 (PMC6232127; doi:10.1038/s41467-018-07235-5)
Supplement: Supplementary file 3 — Description of Additional Supplementary Files [file 41467_2018_7235_MOESM3_ESM.pdf]

Description of Additional Supplementary Files

File Name: Supplementary Data 1
Description: Short-term study mutations compared between in vivo and in vitro conditions

File Name: Supplementary Data 2
Description: Phase switching

File Name: Supplementary Data 3
Description: A list of genes containing 6-14 nucleotide homopolymeric tracts in <i>N. lactamica</i> Y92-1009

File Name: Supplementary Data 4
Description: Detail of single nucleotide polymorphisms occurring during carriage

File Name: Supplementary Data 5
Description: Persistent mutations

File Name: Supplementary Data 6
Description: Isolates selected for genome sequencing

File Name: Supplementary Data 7
Description: List of isolates, volunteer codes, and ENA accession codes
